# Supplementary material for: Local and global chromatin interactions are altered by large genomic deletions associated with human brain development
Source: Nat Commun. 2018 Dec 17;9:5356. doi: 10.1038/s41467-018-07766-x (PMC6297223; doi:10.1038/s41467-018-07766-x)
Supplement: Supplementary file 1 — Supplementary Information [file 41467_2018_7766_MOESM1_ESM.pdf]

Chromatin interactions are altered by large genomic deletions associated with human brain development

Zhang et al.

Supplementary Figures

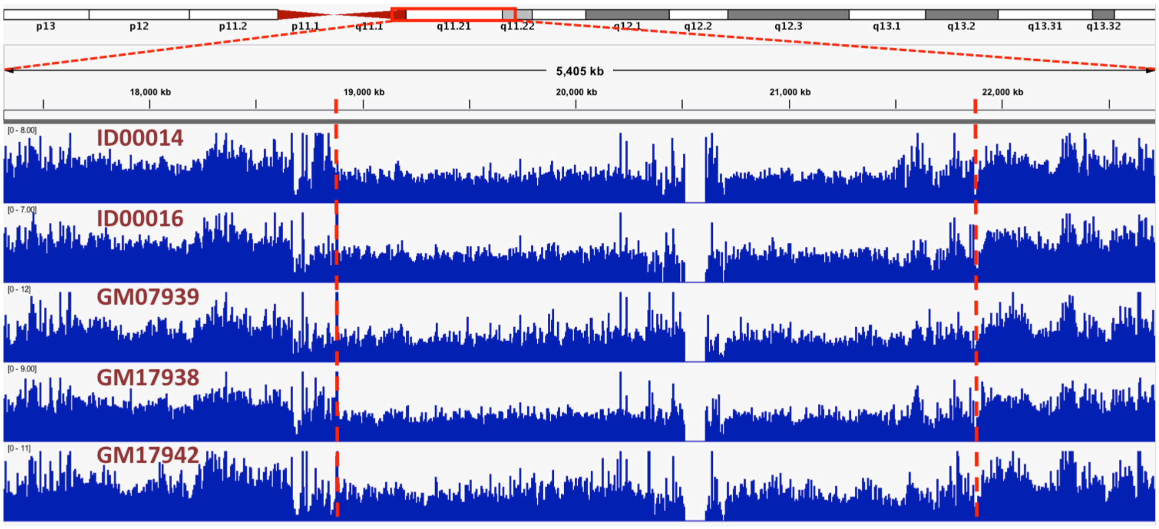

Supplementary Fig. 1. Presence of the 3 Mbp deletion on 22q11.2 in five lymphoblastoid cell lines detected by whole genome sequencing. Each track shows the depth of sequencing read coverage for a given cell line. Red dashed lines mark the boundaries of the deletions. Spikes of read-depth co-localize with regions of segmental duplication (SegDups, also known as LCR regions).

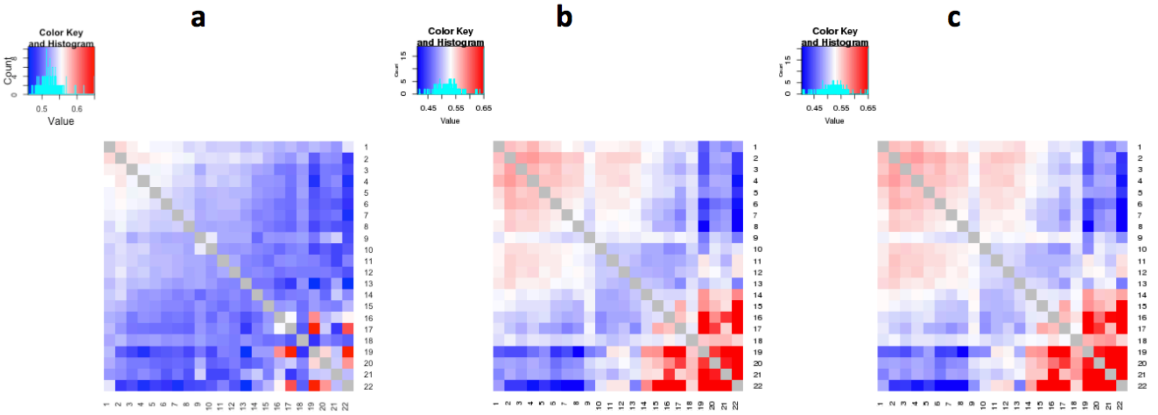

Supplementary Fig. 2. Global maps of interchromosomal contacts after data normalization. (a) Interchromosomal contact map of cell line GM06990 generated by this study. The color scale goes from 0.4 (blue) to 0.55 (white) to 0.7 (red). (b) Interchromosomal contact map of combined control cell lines (n=6). The color scale goes from 0.4 (blue) to 0.525 (white) to 0.65 (red). (c) Interchromosomal contact map of combined 22q11.2del cell lines (n=5). The color scale goes from 0.4 (blue) to 0.55 (white) to 0.7 (red).

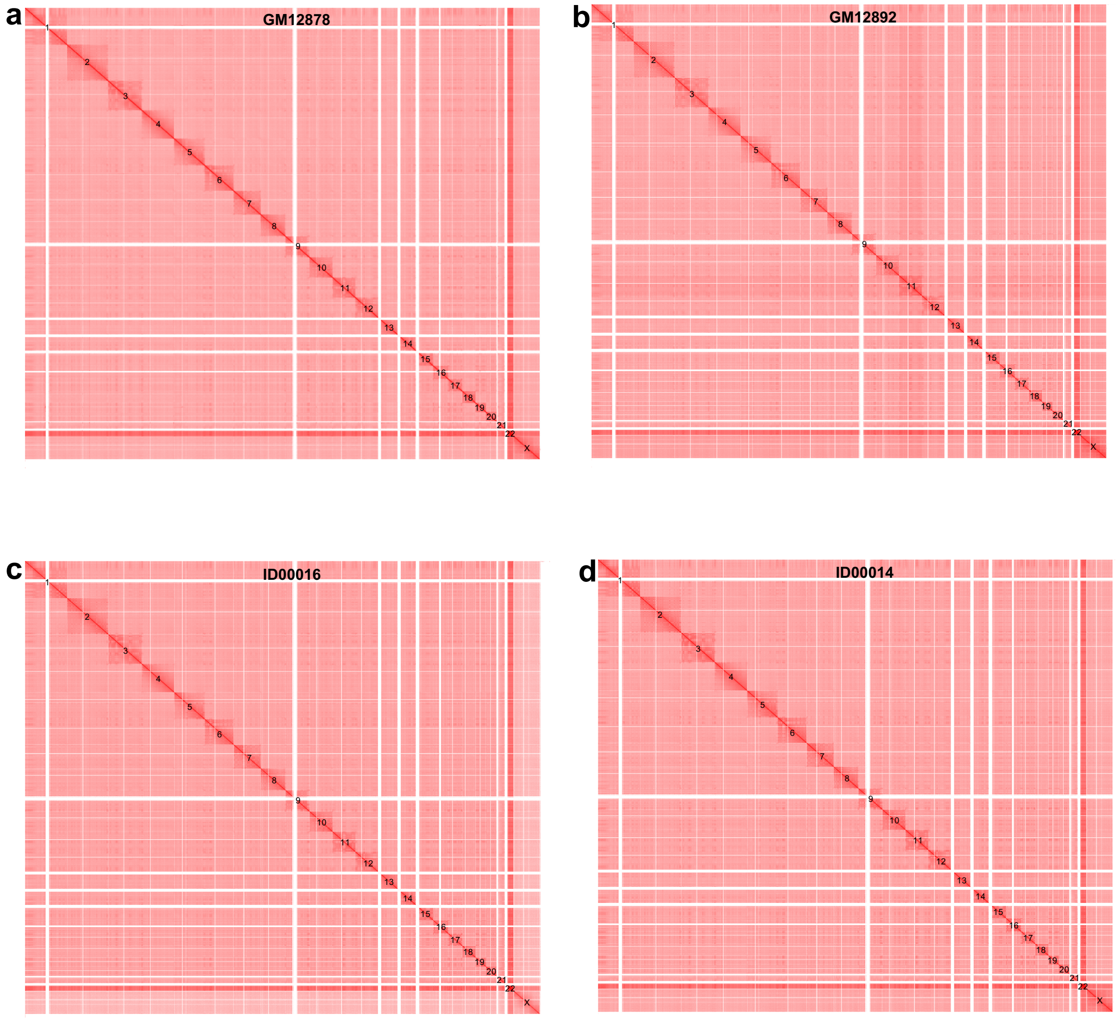

Supplementary Fig. 3. Enrichment of chromosomal contacts involving chromosome 22q in capture Hi-C data. Global chromosomal contacts are shown at 1 Mbp resolution for GM12878 (a), GM12892 (b), ID00016 (c) and ID00014 (d). The plot shows strong enrichment of contacts involving chromosome 22q. As expected there is a background of intra- and inter-chromosomal Hi-C signals not involving chromosome 22q, resulting from incomplete specificity of the targeted capture procedure. The color scale goes from 0 (white) to 14 (red).

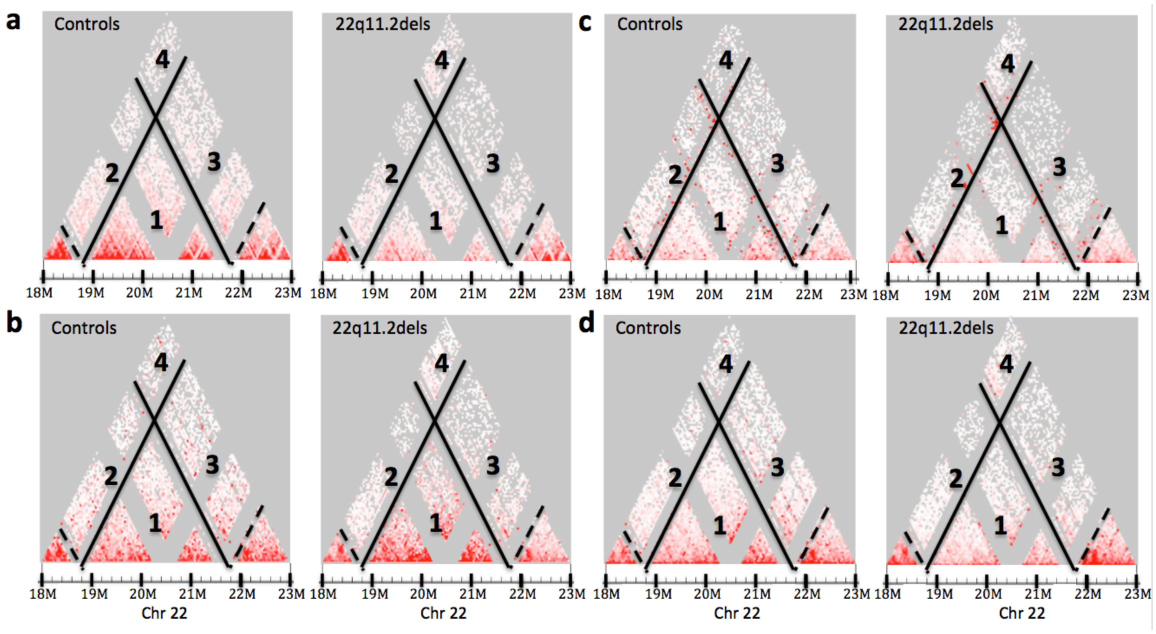

Supplementary Fig. 4. Comparison of different normalization methods. (a) The *cis*-contacts of 22q11.2 regions in raw data without normalization. (b-d) The *cis*-contacts of 22q11.2 regions in metrics normalized by the hiclib package (b), by the HiCNorm package (c), and by the hicpipe package (d). Black dashed lines mark the boundaries for the 22q11.2 deletion. Area 1 shows the contacts within the 22q11.2 deletion region, area 2 and 3 show the contacts between the 22q11.2 deletion and its flanking regions, and area 4 shows the contacts between the flanking regions. Each point represents the contact intensity between two 40 kbp regions. The color scale goes from contacts of low frequency (white) to high frequency (red).

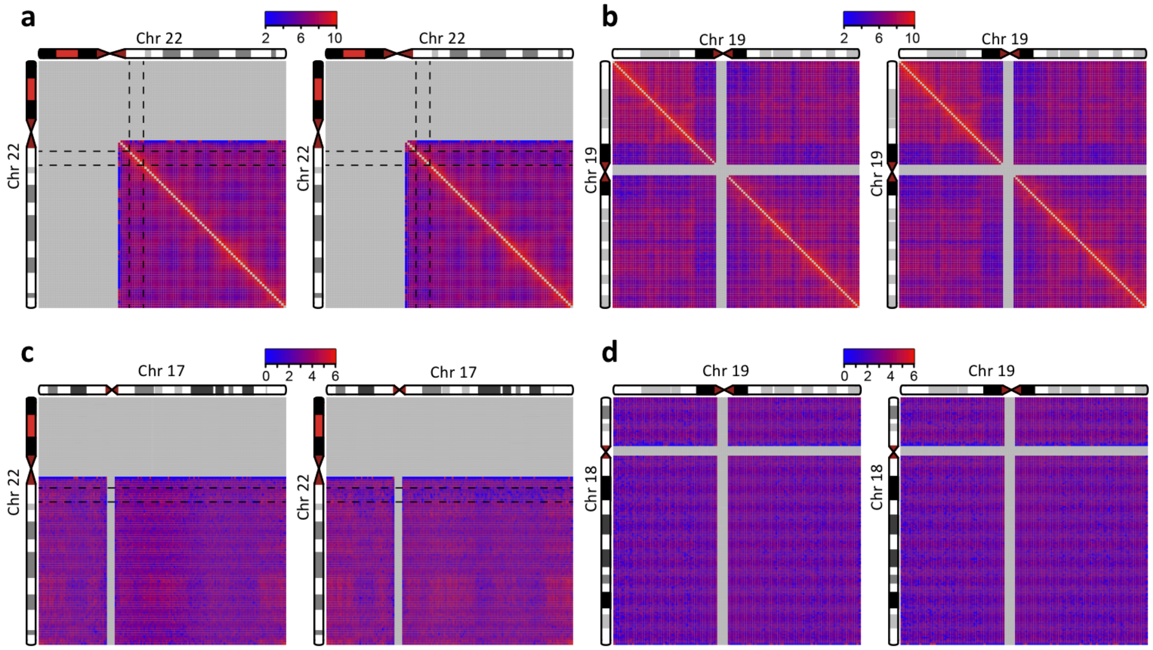

Supplementary Fig. 5. Normalized Hi-C contact maps for selected intra- or inter-chromosomal interaction analyses in controls only or 22q11.2del patients only. Black dashed lines mark the boundaries of the 22q11.2 deletion. Each cell represents the contact between two 500 kbp regions. (a) *Cis*-contacts of chromosome 22 in control cell lines (left, n=6) and in 22q11.2del cell lines (right, n=5). (b) *Cis*-contacts of chromosome 19 in control cell lines (left, n=6) and in 22q11.2del cell lines (right, n=5). (c) *Trans*-contacts between chromosomes 22 and 17 in control cell lines (left, n=6) and 22q11.2del cell lines (right, n=5). (d) *Trans*-contacts between chromosomes 18 and 19 in control cell lines (left, n=6) and 22q11.2del cell lines (right, n=5). The color scale goes from 2 (blue) to 10 (red) in (a-b) and from 0 (blue) to 6 (red) in (c-d).

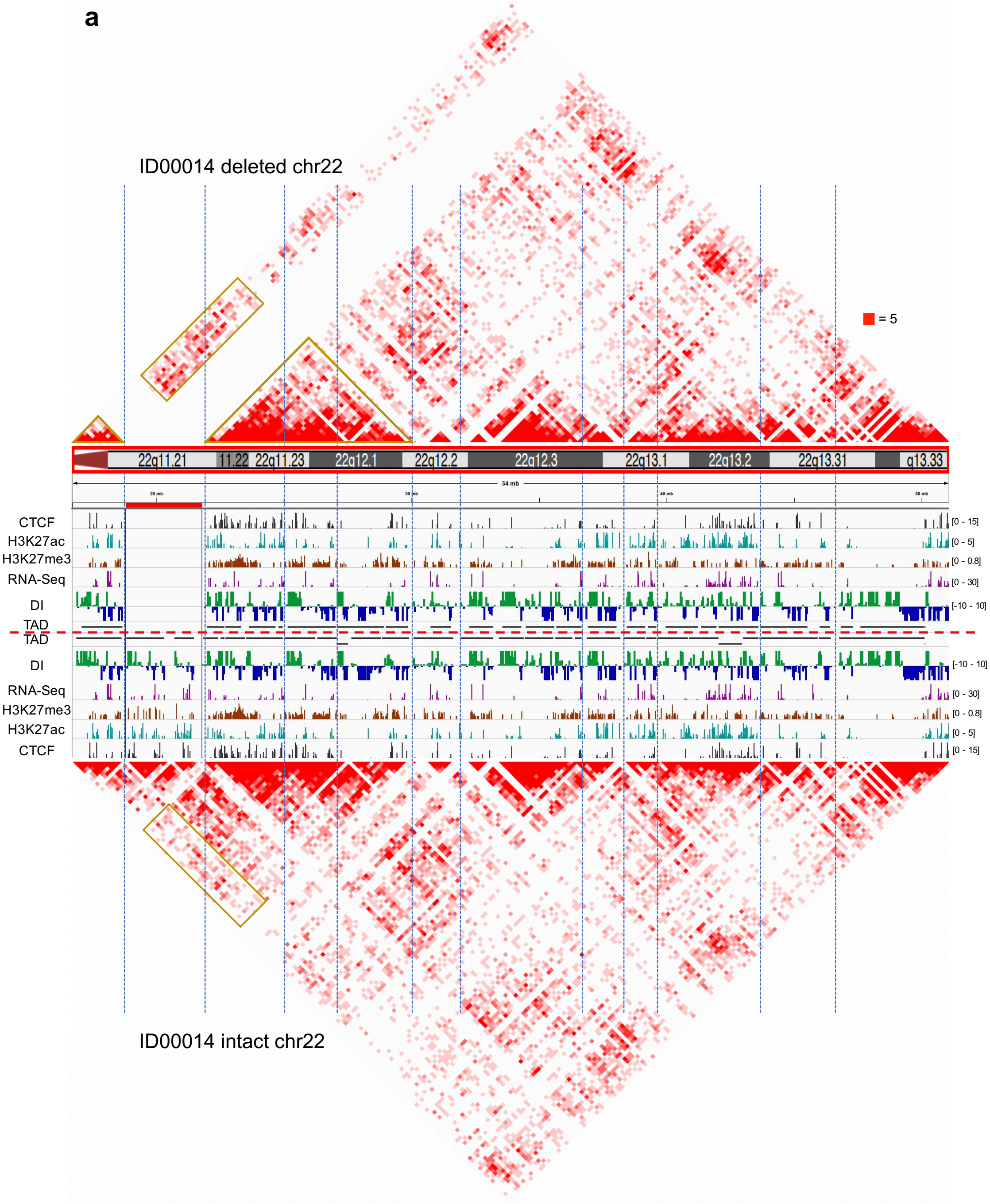
a
ID00014 deleted chr22
= 5
22q11.21
11.22
22q11.23
22q12.1
22q12.2
22q12.3
22q13.1
22q13.2
22q13.31
q13.33
34 mb
CTCF
H3K27ac
H3K27me3
RNA-Seq
DI
TAD
TAD
DI
RNA-Seq
H3K27me3
H3K27ac
CTCF
[0 - 15]
[0 - 5]
[0 - 0.8]
[0 - 30]
[-10 - 10]
ID00014 intact chr22

b

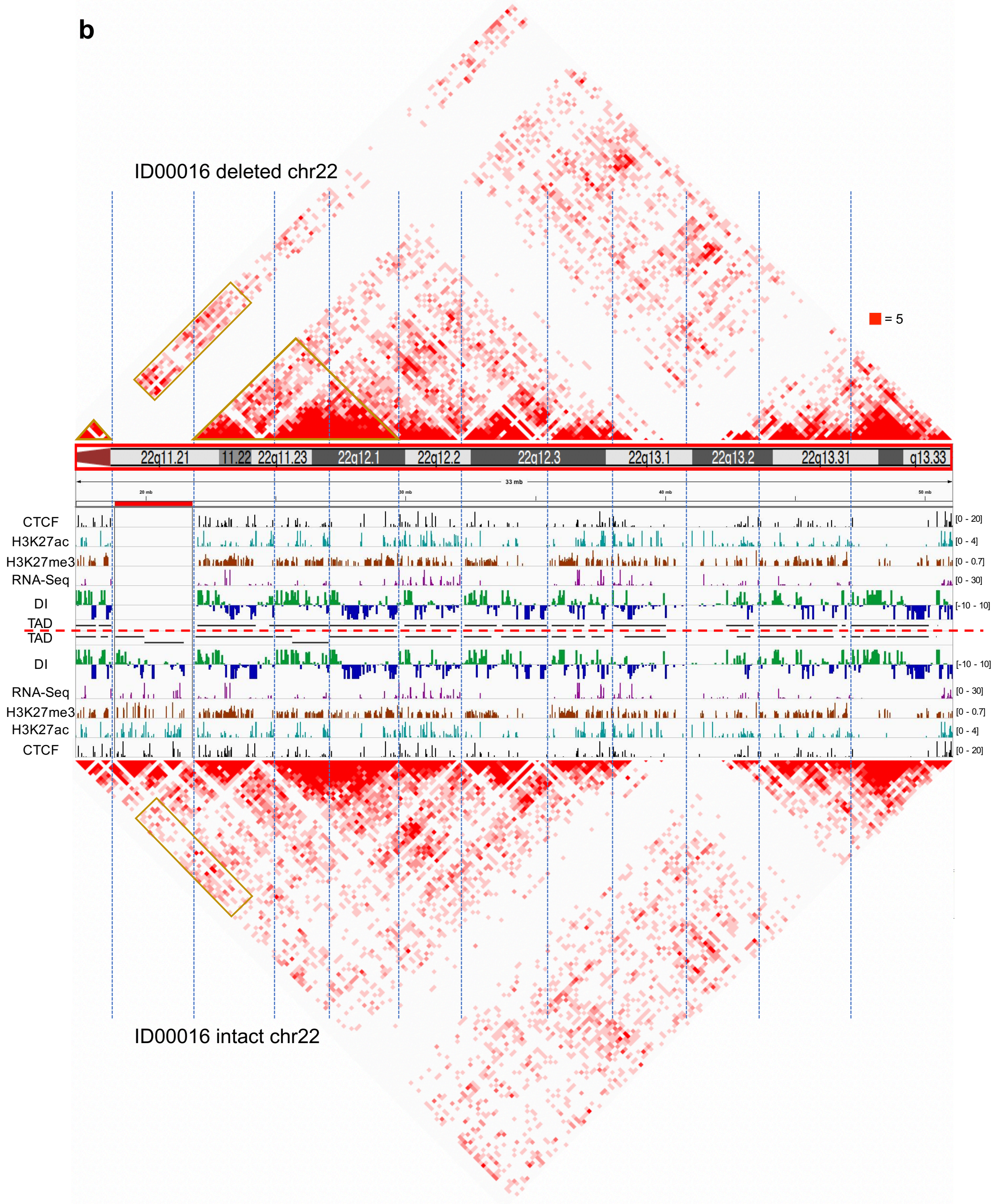
ID00016 deleted chr22
= 5
22q11.21
11.22
22q11.23
22q12.1
22q12.2
22q12.3
22q13.1
22q13.2
22q13.31
q13.33
33 mb
CTCF
H3K27ac
H3K27me3
RNA-Seq
DI
TAD
TAD
DI
RNA-Seq
H3K27me3
H3K27ac
CTCF
[0 - 20]
[0 - 4]
[0 - 0.7]
[0 - 30]
[-10 - 10]
ID00016 intact chr22

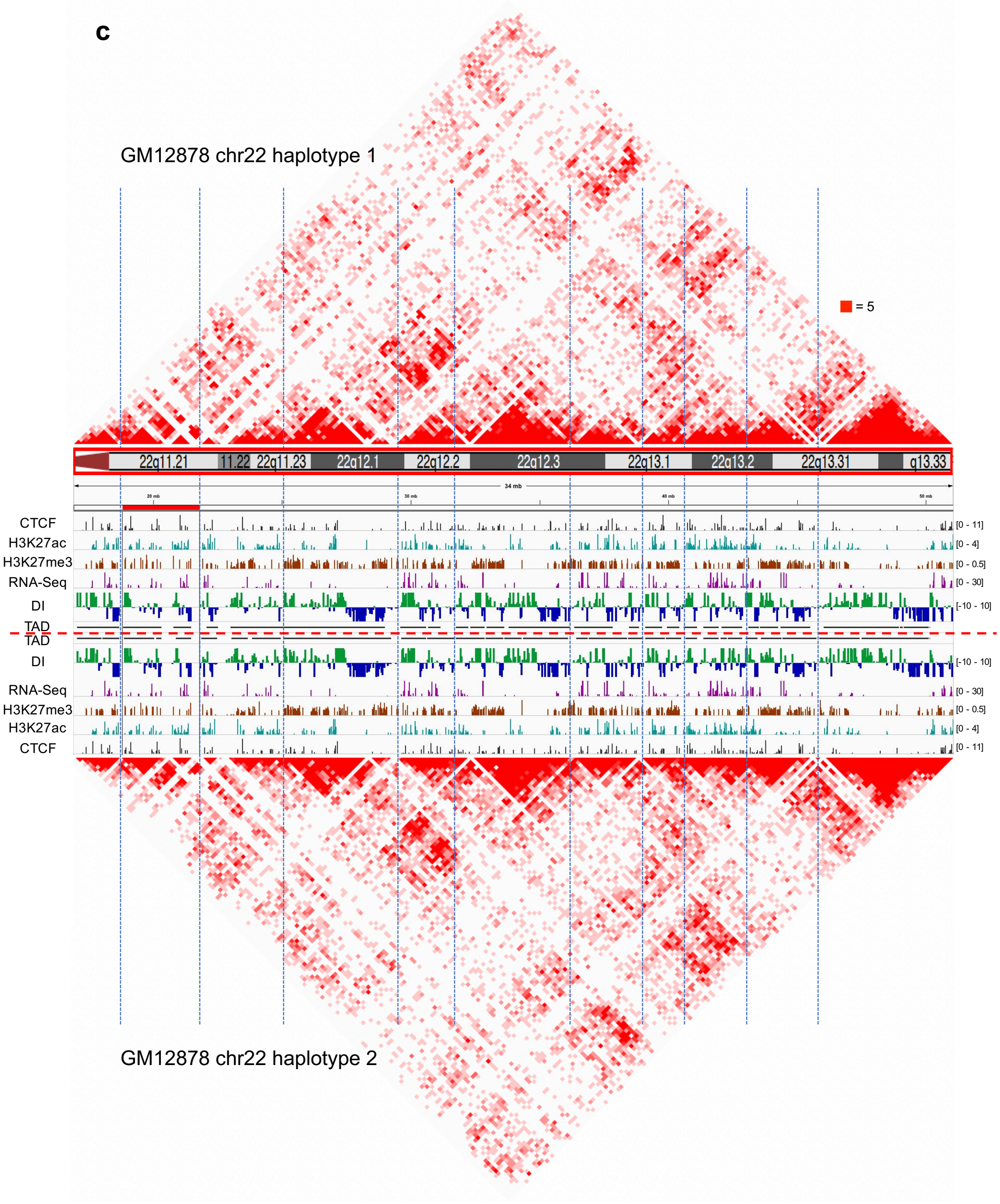
c
GM12878 chr22 haplotype 1
= 5
22q11.21
11.22
22q11.23
22q12.1
22q12.2
22q12.3
22q13.1
22q13.2
22q13.31
q13.33
34 mb
CTCF
H3K27ac
H3K27me3
RNA-Seq
DI
TAD
TAD
DI
RNA-Seq
H3K27me3
H3K27ac
CTCF
[0 - 11]
[0 - 4]
[0 - 0.5]
[0 - 30]
[-10 - 10]
GM12878 chr22 haplotype 2

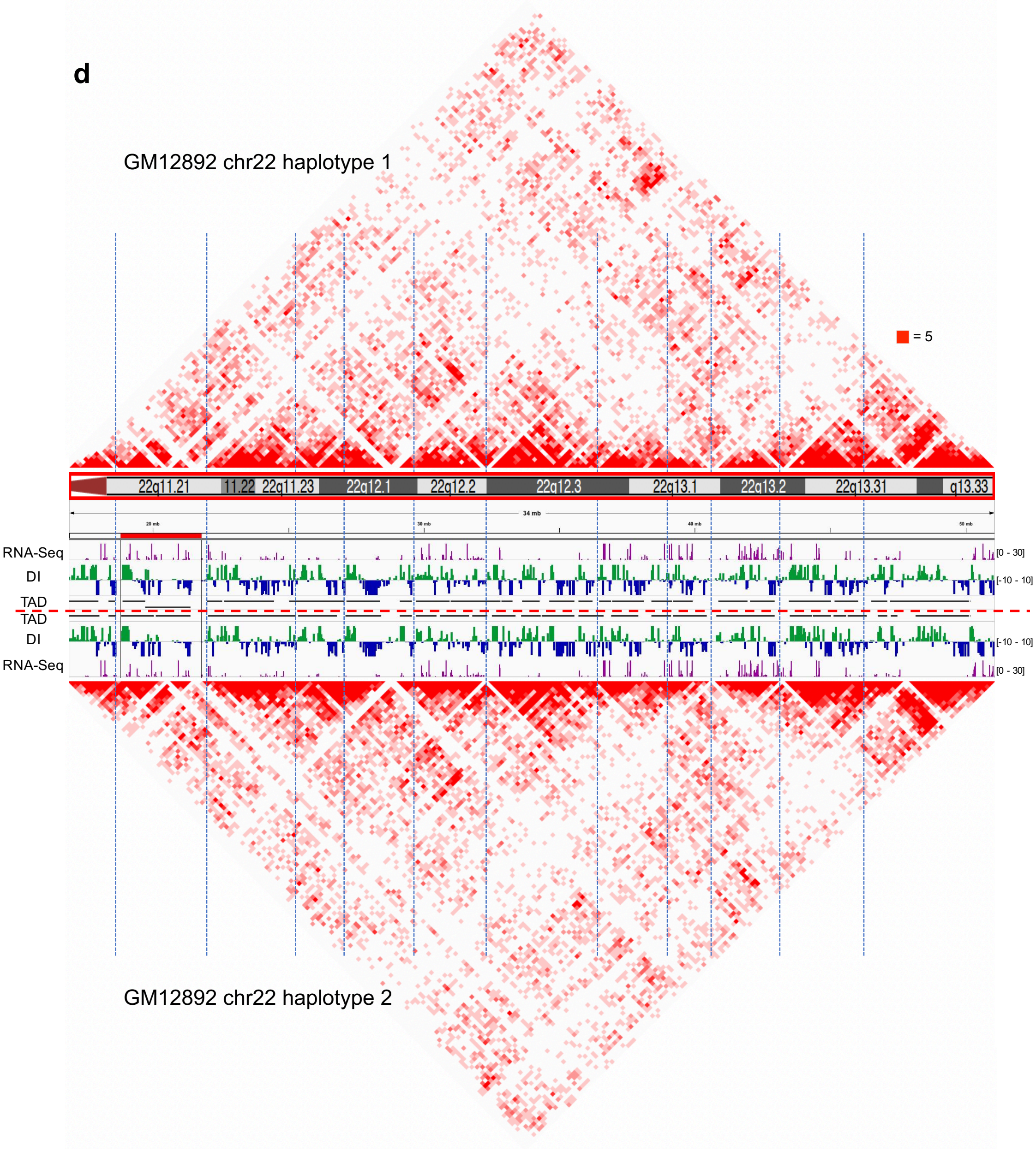

Supplementary Fig. 6. Haplotype-specific contacts and topological domains (TADs) of chromosome 22q. (a) ID00014. (b) ID00016. (c) GM12878. (d) GM12892. The resolution for the heatmaps of chromosomal contacts is 200 kbp**.** The color scale goes from 0 (white) to 5 (red). Direction indexes (DI) calculated at 40 kbp resolution for each homologous chromosome 22q were shown in the middle of each panel. Black lines mark the boundaries of the 22q11.2 deletion. Haplotype-specific gene expression, histone modifications and

CTCF binding are also shown, after normalizing the reads to 10 million read pairs. Red dashed lines separate the two homologous chromosomes 22q. Blue dashed lines mark some of the large topological domains. Yellow rectangle: increased contacts between the topological domains proximal and distal of the 22q11.2 region on the homologous chromosome 22q with the deletion in the patient cell lines. Yellow triangles mark the topological domains involved in the increased contacts.

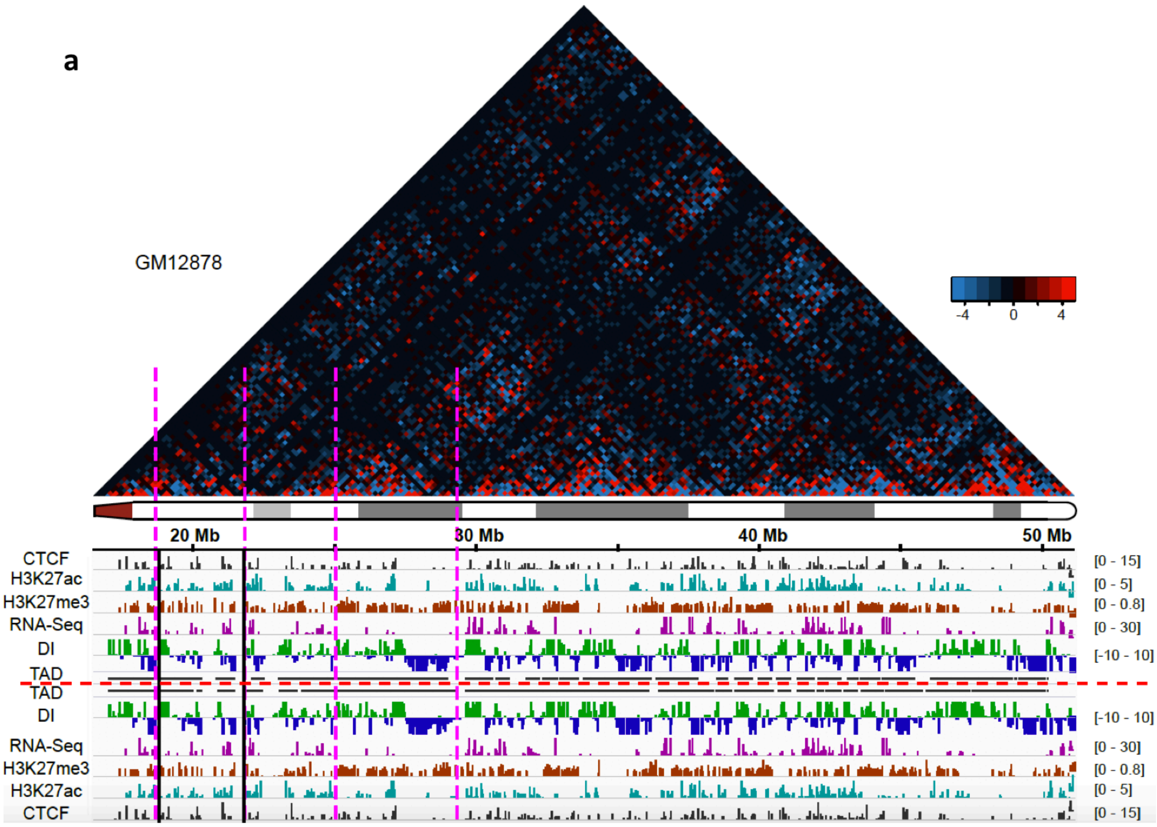
a
GM12878
-4
0
4
20 Mb
30 Mb
40 Mb
50 Mb
CTCF
H3K27ac
H3K27me3
RNA-Seq
DI
TAD
TAD
DI
RNA-Seq
H3K27me3
H3K27ac
CTCF
[0 - 15]
[0 - 5]
[0 - 0.8]
[0 - 30]
[-10 - 10]
[-10 - 10]
[0 - 30]
[0 - 0.8]
[0 - 5]
[0 - 15]

b

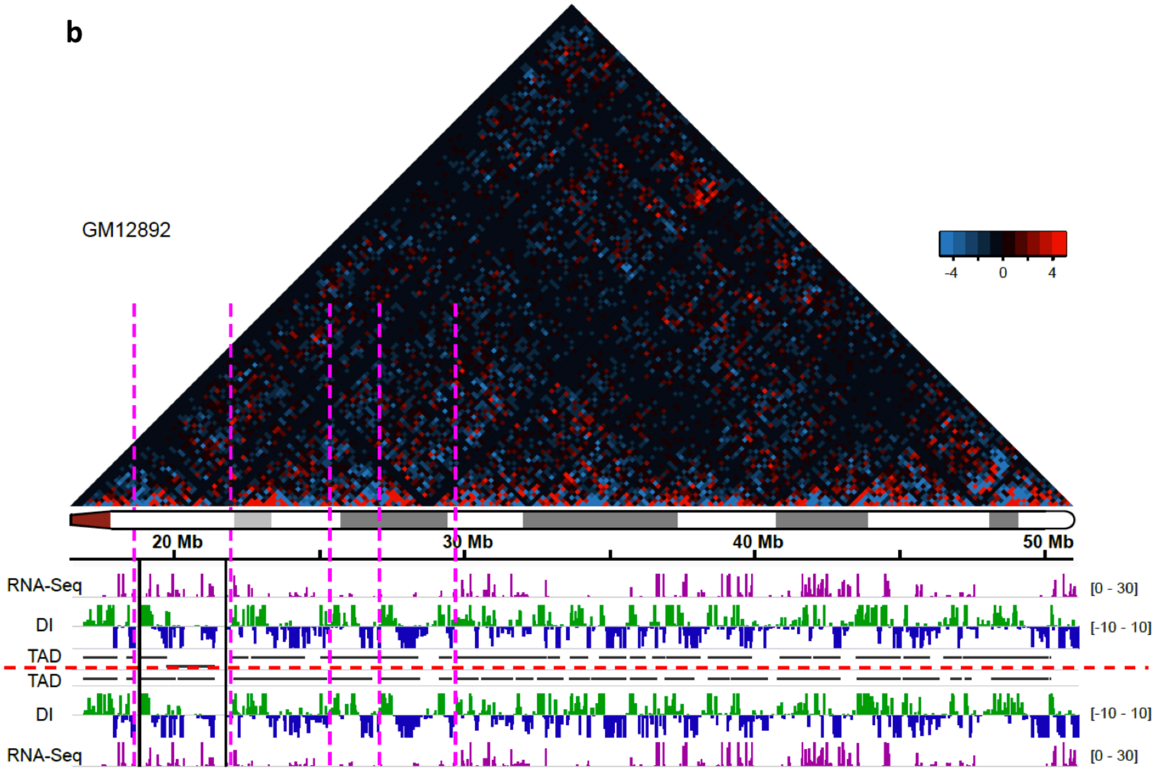

Supplementary Fig. 7. Difference of chromosomal contacts between the two homologous chromosomes 22q and haplotype-specific topological domains for two control cell lines. (a) GM12878. (b) GM12892**.** Shown as heatmaps (200 kbp) are contacts within one homologous chromosome 22q minus those within the other chromosome 22q. The color scale is from -5 (blue) to 5 (red) with 0 represented by black. Direction indexes (DI) are calculated at 40 kbp resolution for each homologous chromosome 22q, shown in the middle of figure. Haplotype-specific gene expression, histone modifications and CTCF binding are shown after normalizing the reads to 10 million read pairs. Red dashed lines separate the tracks for the two homologous chromosomes. Pink dashed lines mark the topological domains involved in the increased contacts on the homologous chromosome with the 22q11.2 deletion in the patient cell lines. Black lines mark the boundaries of the 22q11.2 deletion.

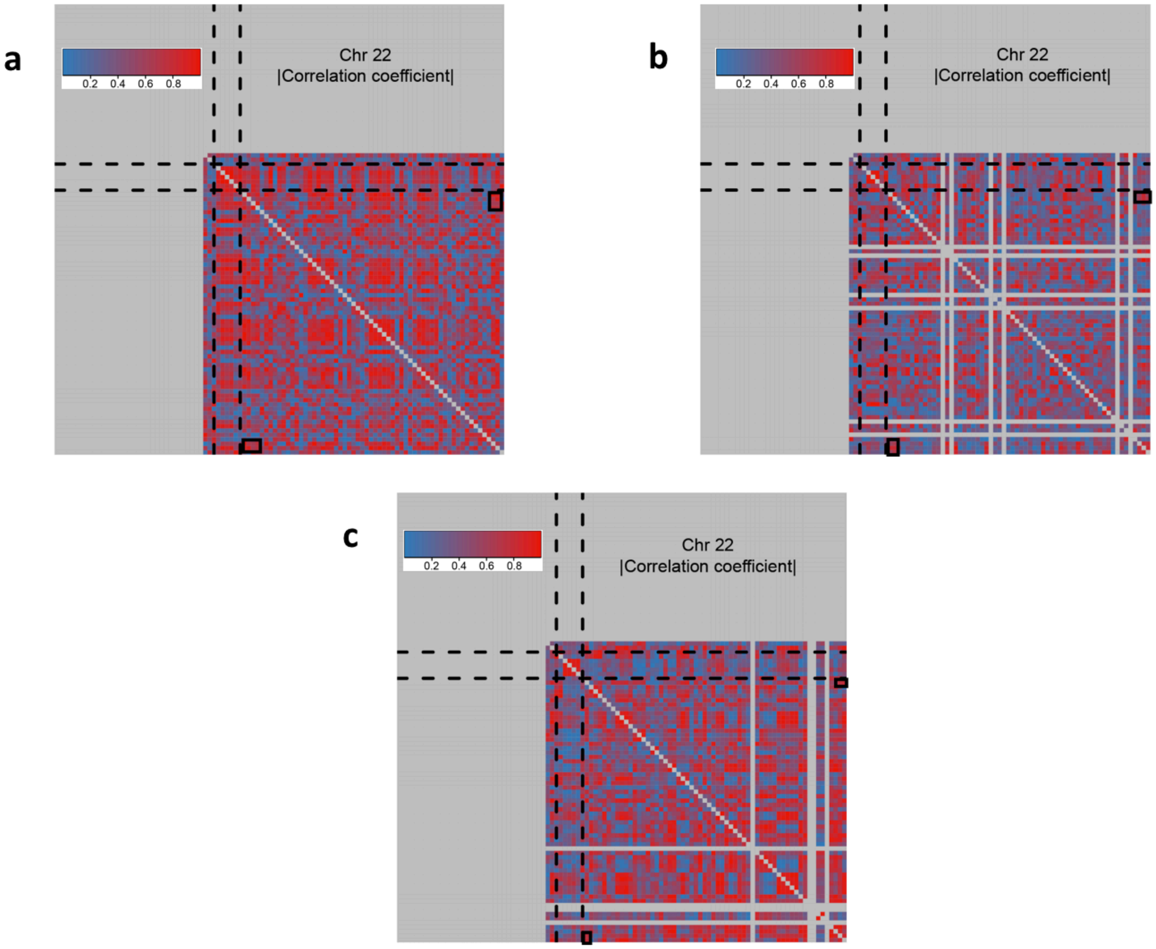

Supplementary Fig. 8. Pearson correlation of ChIP-Seq signals between any two regions on chromosome 22. (a) CTCF binding. (b) H3K27ac marks. (c) H3K27me3 marks**.** Black dashed lines mark the boundaries of the 22q11.2 deletion. Black boxes: correlation between the downstream region of the 22q11.2 deletion and the telomeric region at 50-51 Mbp. The color scale is from 0 (blue) to 1 (red).

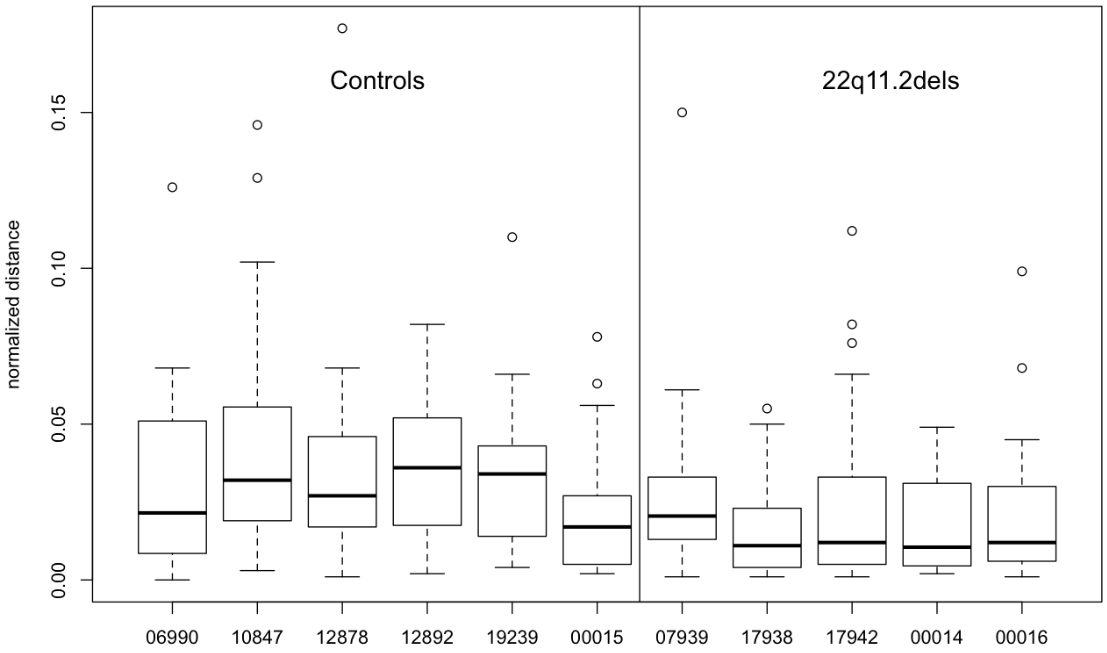

Supplementary Fig. 9. 3D FISH validation of increased contact in the 22q11.2del cells between the downstream boundary region of the 22q11.2 deletion and the telomeric region at 50-51 Mbp. X-axis: cell lines in which 3D FISH was carried out. Y-axis: normalized distance between red and green 3D FISH probes (n=16 to 33 for each cell line). Box represents quartiles, centre line denotes 50th percentile and whiskers extend to the most extreme data points within 1.5 times the interquartile range.

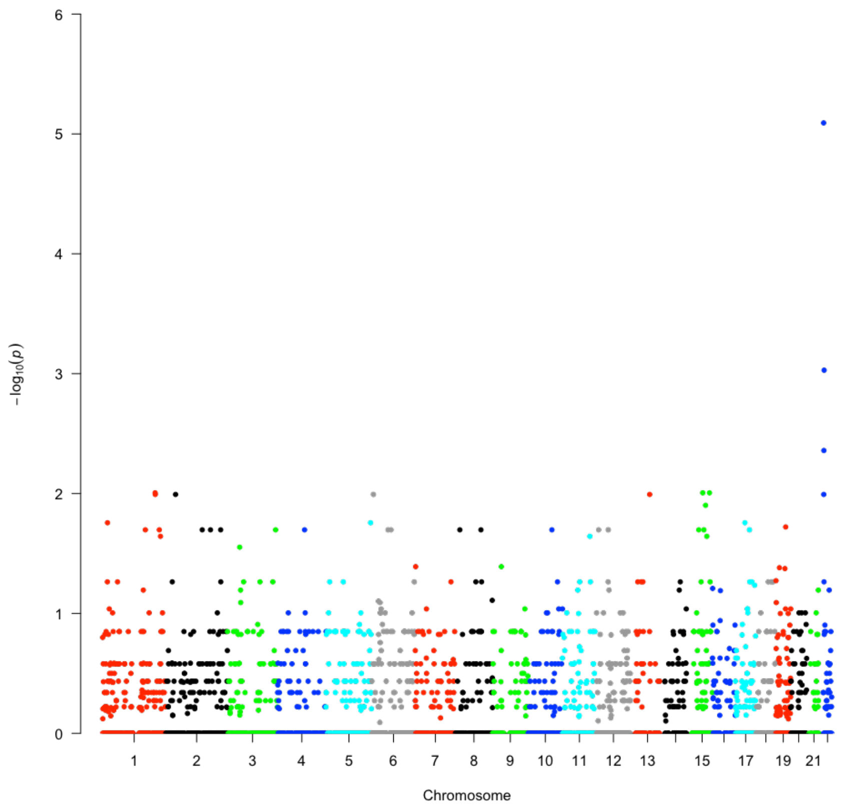

Supplementary Fig. 10. Manhattan plot of whole genome enrichment analysis for differentially expressed genes. -log10 (p values) of Fisher's exact test across the genome are shown. Each dot represents a 500 kbp bin.

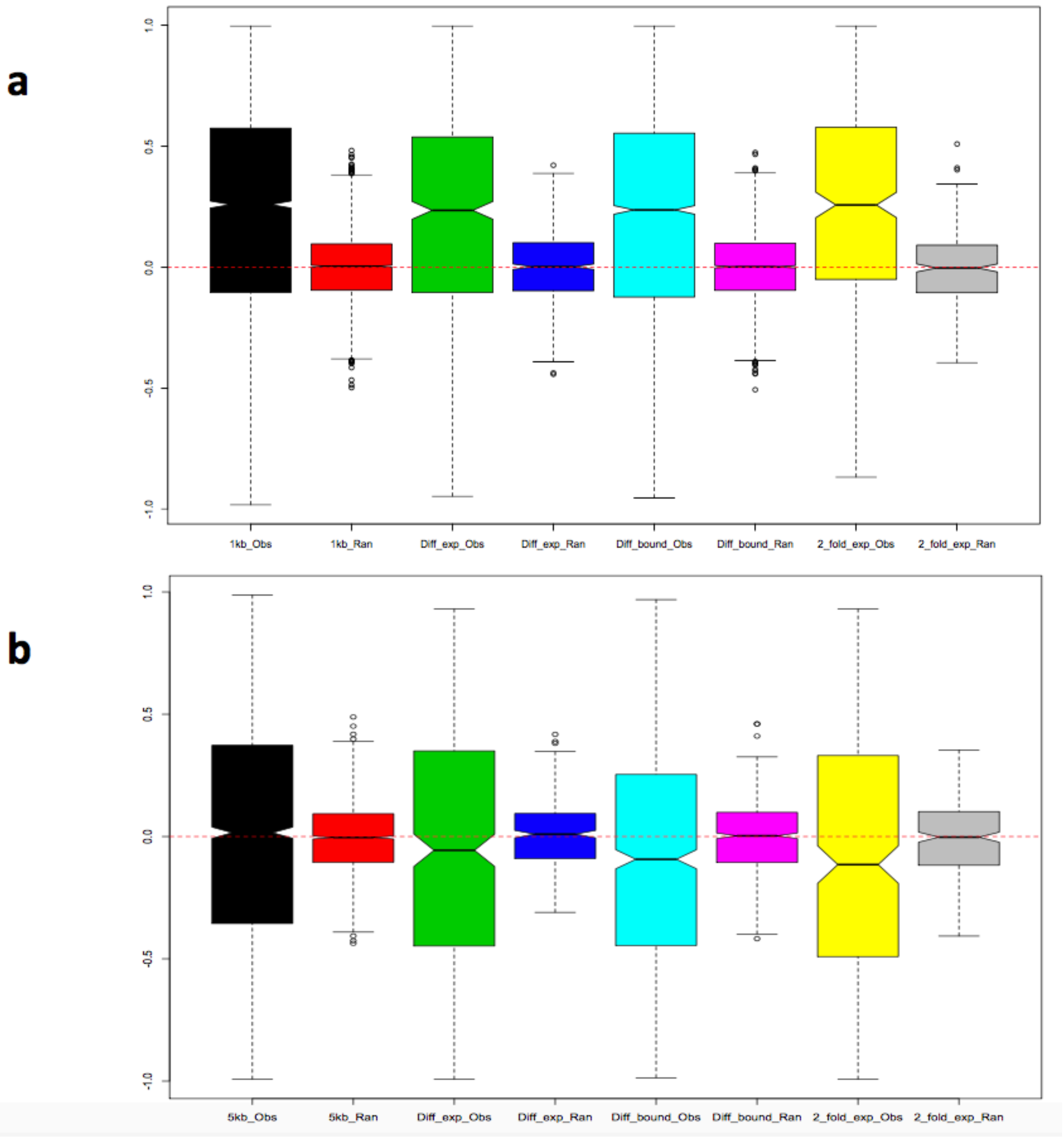

Supplementary Fig. 11. Correlation between gene expression and histone modification across the cell lines. (a) Pearson correlation of gene expression and H3K27ac binding. Only peaks within 1 kbp TSS regions were included in this analysis. 1kb_Obs: observed correlation coefficients between genes' FPKM and H3K27ac binding for all the genes across the individuals; 1kb_Ran: correlation coefficients obtained by 10 permutations using the 1kb_Obs data. Diff_exp_Obs: observed correlation coefficients

between genes’ FPKM and H3K27ac binding for the differentially expressed genes (FDR < 0.05) across the individual cell lines; Diff_exp_Ran: correlation coefficients obtained by 10 permutations using the Diff_exp_Obs data. Diff_bound_Obs: observed correlation coefficients between genes’ FPKM and H3K27ac binding for the genes with differential H3K27ac marks (FDR < 0.05) across the individual cell lines; Diff_bound_Ran: correlation coefficients obtained by 10 permutations using the Diff_bound_Obs data. 2_fold_exp_Obs: observed correlation coefficients between genes’ FPKM and H3K27ac binding for the differentially expressed genes (absolute fold change > 2) across the individual cell lines; 2_fold_exp_Ran: correlation coefficients obtained by 10 permutations using the 2_fold_exp_Obs data. (b) Pearson correlation of gene expression and H3K27me3 binding. Only peaks within 5 kbp TSS regions were included in this analysis. 5kb_Obs: observed correlation coefficients between genes’ FPKM and H3K27me3 binding for all the genes across the individual cell lines; 5kb_Ran: correlation coefficients obtained by 10 permutations using the 5kb_Obs data. Diff_exp_Obs: observed correlation coefficients between genes’ FPKM and H3K27me3 binding for the differentially expressed genes (FDR < 0.05) across the individual cell lines; Diff_exp_Ran: correlation coefficients obtained by 10 permutations using the Diff_exp_Obs data. Diff_bound_Obs: observed correlation coefficients between genes’ FPKM and H3K27me3 binding for the genes with differential H3K27me3 marks (FDR < 0.05) across the individual cell lines; Diff_bound_Ran: correlation coefficients obtained by 10 permutations using the Diff_bound_Obs data. 2_fold_exp_Obs: observed correlation coefficients between genes’ FPKM and H3K27me3 binding for the differentially expressed genes (absolute fold change > 2) across the individual cell lines; 2_fold_exp_Ran: correlation coefficients obtained by 10 permutations using the 2_fold_exp_Obs data. Box represents quartiles, centre line denotes 50th percentile and whiskers extend to the most extreme data points within 1.5 times the interquartile range.

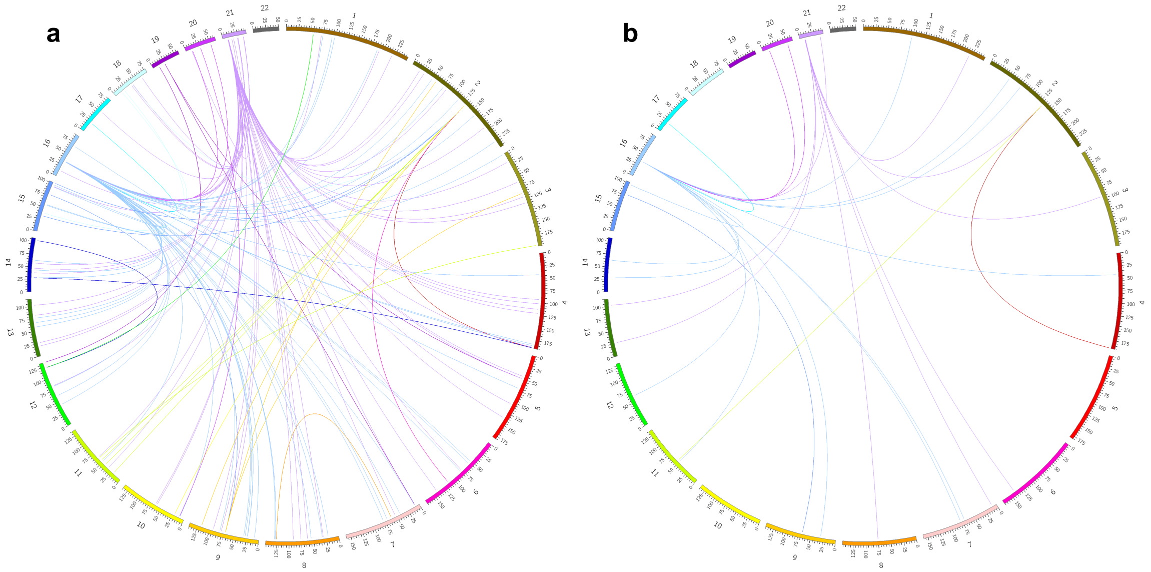

Supplementary Fig. 12. Genome-wide inter-chromosomal contact changes (either more or less frequent) determined by Hi-C analysis in 1q21.1del versus control cell lines. Circos plot of the inter-chromosomal contacts exhibiting differential interaction in 1q21.1del (n=2) versus control (n=6) cell lines at significance level of 1.0E-4 (a) and 1.0E-5 (b) and showing only the top 5% strongest inter-chromosomal contacts.

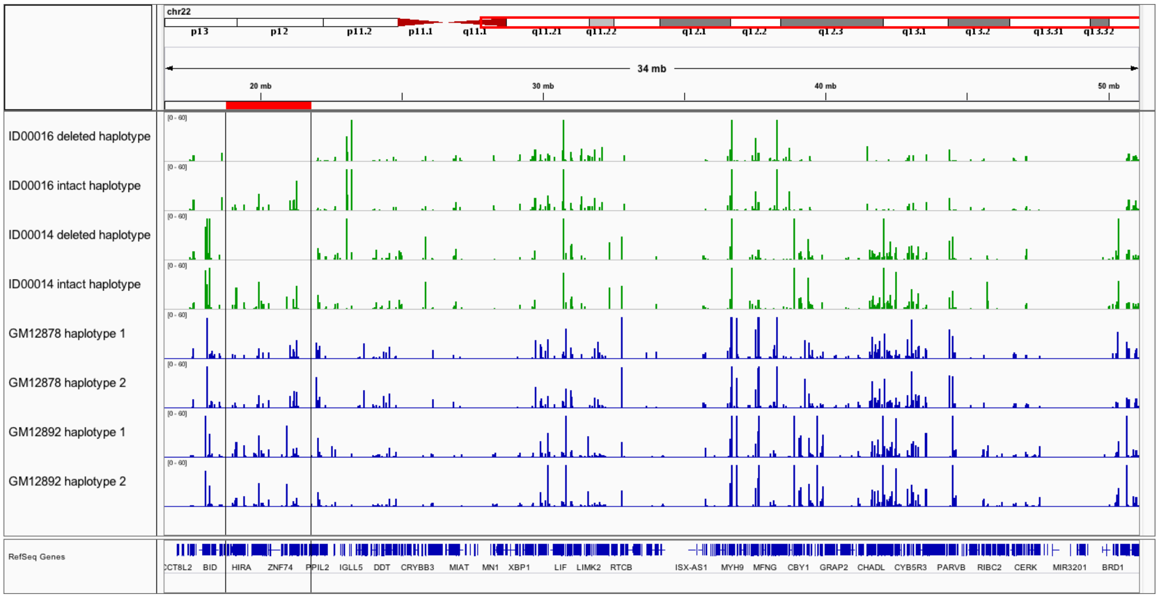

Supplementary Fig. 13. Allele-specific expression (ASE) of genes on chromosome 22q in patient and control cell lines with haplotype phasing and capture Hi-C data. Green is patient cell lines while blue is controls; RNA-Seq read counts were normalized to 10 million read pairs and scaled to the same level; read counts of the 22q11.2 region of the intact chromosome 22q in ID00016 and ID00014 were shown at the same positions of the phased SNVs as in GM12878 and GM12892 respectively. Black lines mark the boundaries of the 22q11.2 deletion. None of the genes on chromosome 22q exhibited ASE only in patient cell lines but not in control cell lines.

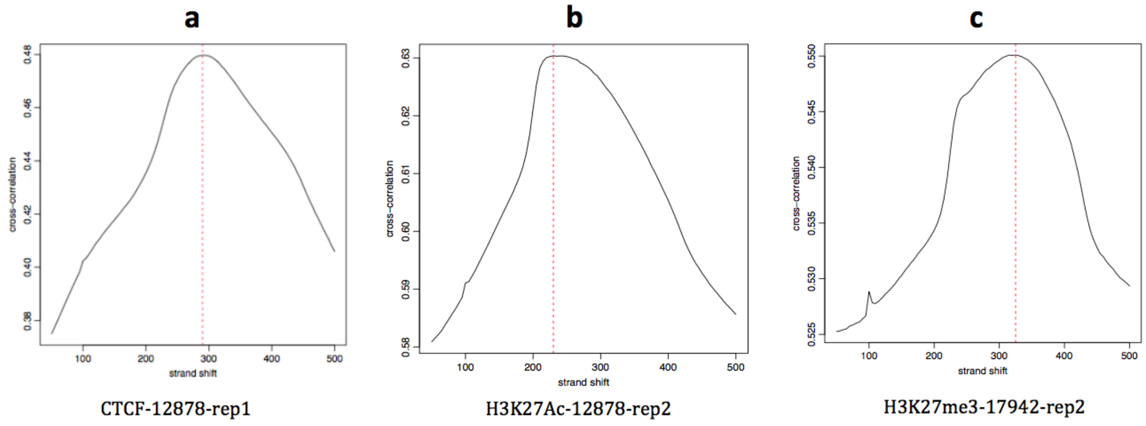

Supplementary Fig. 14. Examples for normalized strand coefficient (NSC) and relative strand correlation (RSC) for assessing signal-to-noise ratios in ChIP-seq experiments. (a) CTCF ChIP-Seq for replicate 2 of GM12878. (b) H3K27ac ChIP-Seq for replicate 2 of GM12878. (c) H3K27me3 ChIP-Seq for replicate 2 of GM17942.

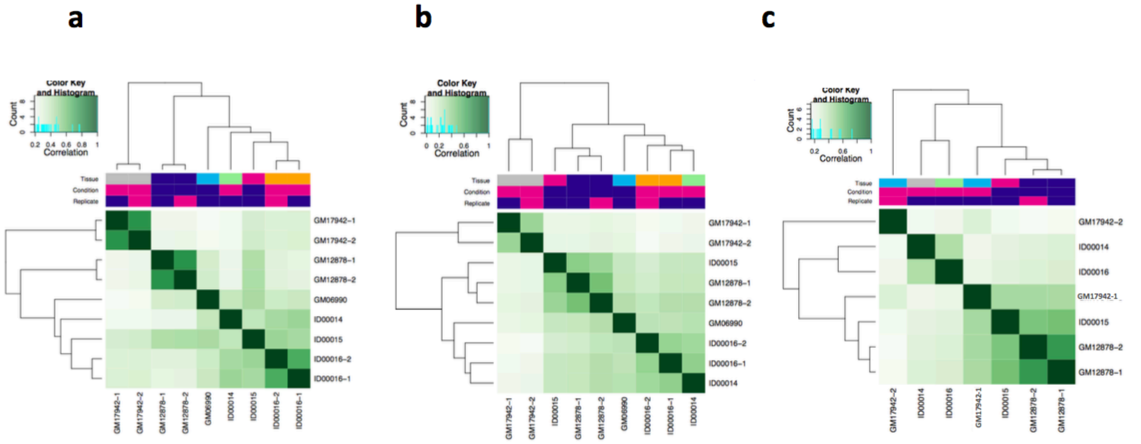

Supplementary Fig. 15. Correlation plots for correlation of binding sites for the ChIP-Seq datasets. (a) Correlation plots for H3K27ac. (b) Correlation plots for H3K27me3. (c) Correlation plots for CTCF. We used a different number of cells for replicate 1 of GM17942.

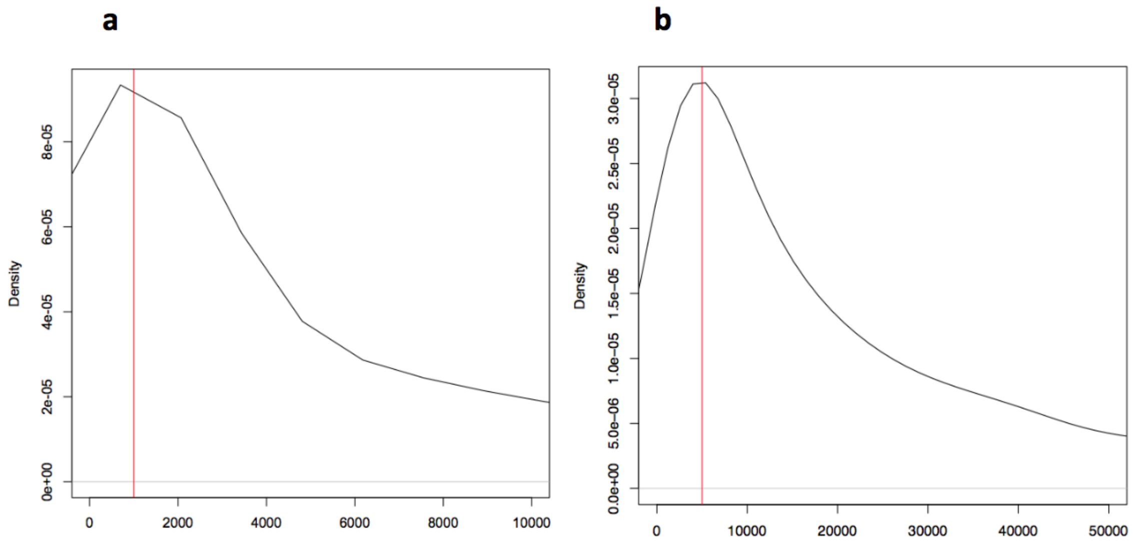

Supplementary Fig. 16. Distribution of the distance between histone mark sites and their nearest TSSs. (a) H3K27ac. (b) H3K27me3**.** Red lines represent the cutoff for distance in which TSSs are associated with the binding sites.

Supplementary Tables

Supplementary Table 1. Hi-C and RNA-Seq datasets generated for this study.

Category	ID	Hi-C Reads Pair Number	Capture Hi-C Reads Pair Number	RNA-Seq Reads Pair Number
Controls	GM06990	119M		126M
	GM12878	406M	354M	57M
	GM12892	404M	359M	51M
	GM18505	116M		55M
	GM10847	167M		52M
	ID00015	161M		83M
	GM19238			62M
	GM19239			53M
	GM19240			54M
22q11.2 deletion	GM17942	206M		93M
	ID00014	363M	393M	89M
	ID00016	353M	350M	104M
	GM07939	420M		77M
	GM17938	198M		29M
1q21.1 deletion	52425	128M		
	82699	130M		

Supplementary Table 2. Genome-wide CNV burden in patient cell lines.

Sample	Number of CNV
ID00014	146
ID00016	148
GM07939	127
GM17938	140
GM17942	120

The number of CNVs called by CNVnator (v0.3.3) [1] for each patient cell line is comparable to the numbers reported by the 1000 Genomes Project (where the median number of CNVs reported per genome is 153-170) [2].

Supplementary Table 3. ChIP-Seq dataset generated for this study.

Category	ID	Replicate	H3K27ac Reads Number	H3K27me3 Reads Number	CTCF Reads Number	Input Reads Number
Controls	GM06990	1	121M	246M		138M
	GM12878	1	112M	163M	120M	127M
	GM12878	2	102M	161M	130M	127M
	ID00015	1		188M	95M	120M
	ID00015	2	105M			138M
22q11.2 deletion	GM17942	1	93M	206M	94M	122M
	GM17942	2	88M	160M	107M	122M
	ID00014	1	114M	217M	125M	118M
	ID00016	1	114M	227M	124M	130M
	ID00016	2	110M	116M		130M

Supplementary Table 4. Permutation test for enrichment of significantly different trans-contacts in the top 5% strongest trans-contacts [see also Figure 6].

Permutation	Top 5% p<0.0001	p<0.0001	Top 5%	total	Fisher's test p value	Fisher's test odds ratio
swap 1	10	243	240,631	4,812,631	0.658	0.823
swap 2	7	240	240,631	4,812,631	0.179	0.583
swap 3	6	271	240,631	4,812,631	0.046	0.443
swap 4	12	268	240,631	4,812,631	0.888	0.896
swap 5	18	298	240,631	4,812,631	0.426	1.208
swap 6	12	261	240,631	4,812,631	0.887	0.920
swap 7	8	240	240,631	4,812,631	0.298	0.667
swap 8	12	272	240,631	4,812,631	0.781	0.882
swap 9	7	278	240,631	4,812,631	0.070	0.504
swap 10	5	257	240,631	4,812,631	0.028	0.389
control 1	19	407	240,631	4,812,631	0.909	0.934
control 2	21	238	240,631	4,812,631	0.018	1.765
control 3	12	195	240,631	4,812,631	0.511	1.231
case 1	10	187	240,631	4,812,631	0.739	1.069
case 2	10	211	240,631	4,812,631	1.000	0.948
case 3	10	210	240,631	4,812,631	1.000	0.952
Real	56	272	240,631	4,812,631	< 2.2e-16	4.119

Swaps 1-10 were performed by randomly swapping the assignment of 22q11.2 deletion and control status across the Hi-C data sets. *Control 1-3* indicates analyses within control cell lines only. *Case 1-3 indicates* analyses within 22q11.2del cell lines. *Real* marks the analysis between actual controls and cases.

Supplementary Table 5. Iterative mapping improved the mapping rate of Hi-C data from below 70% to above 80%.

Sample	GM12878							
	Forward_Reads				Reverse_Reads			
	Total	Uniquely	Multiple	Unmapped	Total	Uniquely	Multiple	Unmapped
Normal mapping	406M	285M (70.19%)	54M (13.43%)	66M (16.37%)	406M	265M (65.41%)	51M (12.73%)	88M (21.85%)
Iterative mapping	406M	351M (86.53%)	45M (11.02%)	9.9M (2.45%)	406M	328M (80.85%)	42M (10.27%)	36M (8.88%)

Sample	GM07939							
	Forward_Reads				Reverse_Reads			
	Total	Uniquely	Multiple	Unmapped	Total	Uniquely	Multiple	Unmapped
Normal mapping	420M	290M (69.07%)	56M (13.21%)	74M (17.72%)	420M	279M (66.39%)	54M (12.87%)	87M (20.74%)
Iterative mapping	420M	363M (86.47%)	46M (10.87%)	11M (2.66%)	420M	350M (83.42%)	44M (10.38%)	26M (6.20%)

Upper table: comparison of mapping rate between normal mapping and iterative mapping for GM12878. Bottom table: comparison of mapping rate between normal mapping and iterative mapping for GM07939.

Supplementary Table 6. Number of chromosomal *cis*- and *trans*-contacts for each sample.

Sample	Raw_cis #	Raw_trans #	Ratio_cis_vs._trans	Filtered_cis #	Filtered_trans #	Ratio_cis_vs._trans
GM18505	27,868,756	38,883,228	0.72	5,413,377	28,437,020	0.19
GM17942	69,229,887	55,819,167	1.24	10,437,784	37,626,347	0.28
GM17938	68,291,638	49,506,346	1.38	12,312,973	36,827,011	0.33
GM10847	52,580,552	44,990,046	1.17	6,326,764	27,512,607	0.23
GM06990	33,008,728	32,444,925	1.02	5,394,994	21,953,940	0.25
ID00015	44,238,248	48,110,469	0.92	7,047,336	28,931,911	0.24
ID00014	47,343,395	60,721,394	0.78	8,360,227	41,198,191	0.2
GM12878	81,706,427	132,818,790	0.62	22,041,738	89,613,166	0.25
GM12892	81,379,308	136,979,103	0.59	30,465,222	101,748,876	0.3
GM07939	83,987,223	152,480,030	0.55	27,843,510	104,363,128	0.27
ID00016	160,874,295	49,003,638	3.28	9,421,904	24,441,032	0.39

"Raw_cis #" and "Raw_trans #" were the number of intra-chromosomal and inter-chromosomal contacts after removing the non-uniquely mapped reads and PCR duplicates in the raw data.

"Filtered_cis #" and "Filtered_trans #" showed the number of read pairs after filtering out self-ligation fragments and the read pairs whose sum of distances from mapped positions to the nearest restriction sites is larger than the length of the fragments in the Hi-C library by hicpipe.

Supplementary Methods

1. Comparison of Hi-C data normalization methods

We compared three different computational normalization methods for Hi-C sequencing data: hiclib [3], hicpipe [4] and HiCNorm [5] and found not all of the methods are robust when applied to Hi-C data from genomes with a large deletion CNV. Compared to the raw *cis*-contacts metrics (**Supplementary Fig. 4a**), the hiclib package did change the *cis*-contacts pattern in the 22q11.2del region (area 1 in **Supplementary Fig. 4b**) while hicpipe and HiCNorm did not over-normalize the *cis*-contacts metrics in the same region (**Supplementary Figs. 4c,d**). Although the contacts between the 22q11.2del region and other regions (area 2 and 3 in **Supplementary Fig. 4a**) were conserved in all of the three metrics, the contacts within the 22q11.2del region (area 1 in **Supplementary Fig. 4**) were normalized to the same level as other regions in hiclib metrics while these contacts remained the same as the raw metrics in the hicpipe and HiCNorm metrics (**Supplementary Fig. 4**).

The hiclib package uses iterative correction for Hi-C data, and thus all of the rows and columns in the contact metrics are generalized to the same value. This correction eliminates the difference between the 22q11.2del region and other regions in the genome. In conclusion our analysis shows that not all of the computational normalization methods are appropriate for Hi-C data from genomes with a large deletion CNV. The computational approach therefore has to be selected with caution, taking the specific characteristics of the genomes in a given study into account. In the current study we used hicpipe as it is robust to the presence of a large deletion CNV.

2. Haplotype phasing

We first performed deep whole genome sequencing of the two patient cell lines from

related donors (437 million read pairs, 37.4x coverage, for ID00014, and 382 million read pairs, 32.8x coverage, for ID00016), on the HiSeq X platform, using paired-end sequencing with a read length of 150 bp. Sequencing reads were mapped to the human reference genome (hg19) using Isaac [6] and single nucleotide variants (SNVs) were called by Isaac Variant Caller (2.0.13). In total, 2,143,593 and 2,152,457 heterozygous SNVs were called for ID00014 and ID00016, respectively.

As ID00014 is a parent of ID00016 we were able to phase 1,085,348 and 1,096,129 heterozygous SNVs for ID00014 and ID00016, respectively, on the chromosome level based on Mendelian inheritance. However, Mendelian inheritance can only phase SNVs that are heterozygous in one individual and homozygous in the other. SNVs that are heterozygous in both ID00014 and ID00016 cannot be phased.

To phase more heterozygous SNVs, we conducted statistically aided, long-read haplotyping (SLRH) [7]. Genomic DNA was sheared into fragments of about 10 kbp, diluted and distributed into a 384-well plate. Each well contains 3,000–6,000 molecules which ensures that almost never the same locus will be present twice or more in a given well, which allows the fragments within each well to be assembled after sequencing. Fragments within each well were amplified, cut into short fragments and barcoded. Each well has a unique barcode, giving all the fragments from one well the same barcode. Fragments from all wells were pooled together and sequenced on Illumina HiSeq2000 sequencers using2x100 bp paired-end sequencing. The number of read pairs obtained was 81 million for ID00014 and 79 million for ID00016.

Sequencing reads were aligned to the reference genome using BWA (v0.7.5a) [8] and assigned to their original well based on the barcode specific to each well. For each well reads were assembled at their overlapping heterozygous SNVs into haplotype blocks. Then

these haplotype blocks were assigned to long haplotype contigs statistically based on a phased reference panel using Illumina's TruSeq phasing analysis tool Prism v2.2. However, the haplotype contigs obtained by this method cannot be connected on the whole chromosome level without pedigree structure information.

To assemble these haplotype contigs into whole chromosome, SNVs phased by Mendelian inheritance were assigned to the phased haplotype contigs obtained above. Within each haplotype contig, SNVs that are heterozygous in both ID00014 and ID00016 were phased on the whole chromosome level based on the SNVs phased by Mendelian inheritance in the same haplotype contig. If multiple SNVs phased by Mendelian inheritance exist within one halotype contig, SNVs that are heterozygous in both ID00014 and ID00016 can be phased by multiple SNVs. We require > 80% consistency for a SNV that is heterozygous in both ID00014 and ID00016 to be phased on the whole chromosome level. In total, 790,650 and 781,814 SNVs heterozygous in both ID00014 and ID00016 were phased on the chromosome level at this step for ID00014 and ID00016, respectively.

After the above chromosome level phasing, some SNVs heterozygous in both ID00014 and ID00016 might only be phased in one individual. Mendelian inheritance was applied again to these SNVs and 6,560 and 7,247 SNVs were phased on the chromosome 22q level for ID00014 and ID00016, respectively. Also, we removed the SNVs that were not consistent with Mendelian inheritance from both individuals.

Taken together, 1,868,316 and 1,870,948 heterozygous SNVs on autosomes were phased on the chromosome level for ID00014 (87.2%) and ID00016 (87.0%) respectively.

To obtain haplotype phasing data of control samples, phased vcf files of a trio — GM12878, GM12891 and GM12892 — were downloaded from public databases [7]. These

three samples had been whole-genome sequenced to a depth of ~50x by the Illumina Platinum Genomes Project (http://www.illumina.com/platinumgenomes/) and also had been haplotype-phased by statistically aided, long-read haplotyping (SLRH) [7]. As we generated capture Hi-C data for GM12878 and GM12892, we conducted the same phasing analysis on them as on the two patient samples except that GM12891 was also used to increase the number of phased SNVs when phasing the SNVs on the chromosome level by Mendelian inheritance. In total, 1,929,967 and 1,874,181 heterozygous SNVs on autosomes were phased on the chromosome level for GM12892 (98.5%) and GM12878 (98.4%) respectively.

Supplementary References

1 Abyzov, A., Urban, A. E., Snyder, M. & Gerstein, M. CNVnator: an approach to discover, genotype, and characterize typical and atypical CNVs from family and population genome sequencing. *Genome Res* **21**, 974-984, doi:10.1101/gr.114876.110 (2011).

2 Auton, A. *et al.* A global reference for human genetic variation. *Nature* **526**, 68-74, doi:10.1038/nature15393 (2015).

3 Imakaev, M. *et al.* Iterative correction of Hi-C data reveals hallmarks of chromosome organization. *Nat Methods* **9**, 999-1003, doi:10.1038/nmeth.2148 (2012).

4 Yaffe, E. & Tanay, A. Probabilistic modeling of Hi-C contact maps eliminates systematic biases to characterize global chromosomal architecture. *Nat Genet* **43**, 1059-1065, doi:10.1038/ng.947 (2011).

5 Hu, M. *et al.* HiCNorm: removing biases in Hi-C data via Poisson regression. *Bioinformatics* **28**, 3131-3133, doi:10.1093/bioinformatics/bts570 (2012).

6 Raczy, C. *et al.* Isaac: ultra-fast whole-genome secondary analysis on Illumina sequencing platforms. *Bioinformatics* **29**, 2041-2043, doi:10.1093/bioinformatics/btt314 (2013).

7 Kuleshov, V. *et al.* Whole-genome haplotyping using long reads and statistical methods. *Nat Biotechnol* **32**, 261-266, doi:10.1038/nbt.2833 (2014).

8 Li, H. & Durbin, R. Fast and accurate short read alignment with Burrows-Wheeler transform. *Bioinformatics* **25**, 1754-1760, doi:10.1093/bioinformatics/btp324 (2009).
